# Heterogeneity in Heart Failure with Preserved Ejection Fraction: A Systematic Review of Phenotypic Classifications and Clinical Implications

**DOI:** 10.3390/jcm14144820

**Published:** 2025-07-08

**Authors:** Francisco Epelde

**Affiliations:** Internal Medicine Department, Parc Taulí Hospital Universitari, Institut d’Investigació i Innovació Parc Taulí (I3PT-CERCA), Universitat Autònoma de Barcelona, 08208 Sabadell, Spain; fepelde@gmail.com

**Keywords:** heart failure with preserved ejection fraction, phenotyping, machine learning, precision medicine, cardiovascular heterogeneity

## Abstract

**Background/Objectives:** Heart failure with preserved ejection fraction (HFpEF) has emerged as one of the most challenging syndromes in modern cardiology due to its complex pathophysiology, diagnostic ambiguity, and lack of effective targeted therapies. Unlike heart failure with reduced ejection fraction (HFrEF), HFpEF encompasses a highly heterogeneous patient population unified only by a preserved left ventricular ejection fraction (LVEF) ≥ 50%. This broad definition overlooks important biological and clinical differences, leading to inconclusive results in large-scale therapeutic trials and suboptimal patient outcomes. In recent years, advances in data-driven methodologies—such as unsupervised machine learning, cluster analysis, and latent class modeling—have enabled the identification of distinct HFpEF phenotypes. These phenotypes, often defined by demographic, clinical, hemodynamic, and biomarker profiles, exhibit differential prognoses and treatment responses. **Methods:** This systematic review synthesizes findings from 20 studies published between 2010 and 2025, examining phenotypic classification strategies and their clinical implications. **Results:** Despite methodological variation, several recurring phenotypes emerge, including metabolic–obese, frail–elderly, atrial-fibrillation-dominant, cardiorenal, and pulmonary hypertension/right-heart phenotypes. Each presents a distinct pathophysiological mechanism and risk profile, highlighting the inadequacy of current one-size-fits-all treatment approaches. The review also explores the prognostic value of phenotypes, the impact of phenotypic variation on treatment efficacy, and the methodological challenges that hinder translation into clinical practice—such as inconsistent input variables, lack of external validation, and limited integration with real-world data. **Conclusions:** Ultimately, the findings underscore the need for a paradigm shift from ejection fraction-based classification to phenotype-guided management in HFpEF. Embracing a precision medicine framework could enable personalized treatment strategies, improve clinical trial design, and enhance outcomes for this diverse patient population. The review concludes by outlining future directions, including the development of standardized phenotyping algorithms, integration of multi-omic and digital health data, and the implementation of pragmatic, phenotype-stratified clinical trials.

## 1. Introduction

Heart failure with preserved ejection fraction (HFpEF) has emerged over the past two decades as one of the most pressing and elusive challenges in cardiovascular medicine [1]. Accounting for more than 50% of all heart failure (HF) hospitalizations in developed countries, its prevalence is steadily increasing in parallel with aging populations and the global rise in comorbid conditions such as obesity, diabetes mellitus, hypertension, and atrial fibrillation [2]. Despite its growing impact, HFpEF remains a syndrome characterized by diagnostic ambiguity, therapeutic uncertainty, and a persistently poor prognosis [3]. Hospital readmission rates and long-term mortality in HFpEF are comparable to those seen in heart failure with reduced ejection fraction (HFrEF), yet the former has not benefited from the same advances in disease-modifying therapies [4].

The term “HFpEF” was formally introduced in the early 2000s as a refinement of “diastolic heart failure”, which inadequately captured the range of pathophysiological mechanisms involved. Although the current definition—clinical signs and symptoms of HF with a left ventricular ejection fraction (LVEF) ≥50%—is practical for broad classification, it fails to reflect the complexity underlying this condition [5]. The preserved ejection fraction in HFpEF belies the significant alterations in cardiac structure and function that occur [6], including impaired left ventricular relaxation and compliance, increased left atrial pressure, chronotropic incompetence, endothelial dysfunction, and systemic inflammation [7]. Furthermore, HFpEF frequently coexists with extracardiac abnormalities such as renal dysfunction, pulmonary hypertension, and skeletal muscle wasting, suggesting a multisystemic disorder rather than a purely cardiac pathology [8,9].

A major limitation of the current diagnostic paradigm is its reliance on a single numerical threshold—LVEF ≥ 50%—as a defining feature [10]. This arbitrary cutoff does not account for the gradation of functional impairment across the heart failure spectrum and obscures meaningful biological differences among patients [11]. More importantly, it groups together a highly heterogeneous population under a single diagnostic label, complicating both clinical management and trial design [12]. In recent years, several studies employing machine learning, cluster analysis, and latent class modeling have provided compelling evidence for the existence of distinct phenotypes within HFpEF. These phenotypes—often defined by demographic factors, comorbidity patterns, hemodynamic features, or biomarker profiles—demonstrate varying clinical trajectories and differential responses to therapy [13,14].

This conceptual shift has profound implications for clinical care and research. The failure of multiple large randomized controlled trials (RCTs) to demonstrate robust benefits of traditional HF therapies in HFpEF may be, in part, attributable to this underlying heterogeneity [15]. Trials such as I-PRESERVE, CHARM-Preserved, and TOPCAT failed to show consistent mortality or morbidity benefits [16], despite biological plausibility and prior success in HFrEF populations. More recently, trials with sodium–glucose cotransporter 2 inhibitors (SGLT2is) such as EMPEROR-Preserved and DELIVER have shown promising results, yet their overall effect sizes remain modest. It is increasingly apparent that a “one-size-fits-all” therapeutic strategy is unlikely to succeed in HFpEF [16,17,18].

Recognizing HFpEF as a syndrome composed of multiple overlapping or coexisting pathophysiological processes offers a more nuanced framework. For example, one patient may present with HFpEF primarily driven by metabolic dysfunction and systemic inflammation [19], while another may be primarily affected by concentric remodeling secondary to long-standing hypertension [20]. A third patient might exhibit a predominantly right-sided phenotype due to pulmonary hypertension or significant atrial fibrillation burden [11]. Although these patients share the same LVEF and similar symptoms, the mechanisms driving their disease—and, therefore, the optimal treatment approaches—are markedly different [21].

Several phenotype classifications have been proposed in the literature, including those based on comorbidity clusters (e.g., metabolic, cardiorenal, pulmonary), demographic factors (e.g., elderly frail women vs. middle-aged patients with hypertension), or hemodynamic profiles (e.g., high-output HFpEF, preload-dependent HFpEF). While these classifications have enriched our understanding, there is currently no universally accepted schema [22]. Moreover, the methodological heterogeneity across studies—ranging from the choice of variables for clustering to the statistical techniques employed—limits direct comparison and hinders translation into clinical practice [23].

The aim of this systematic review is to critically examine the current evidence regarding phenotypic heterogeneity in HFpEF. Specifically, we seek to (1) identify and describe the different phenotypes proposed in the literature; (2) evaluate the methodologies used to derive these phenotypes; (3) assess the clinical relevance of each phenotype in terms of prognosis, therapeutic response, and pathophysiological insight; and (4) discuss the potential of a phenotype-driven approach to transform HFpEF management. In doing so, we hope to clarify whether HFpEF should continue to be treated as a singular entity or redefined as a syndrome composed of multiple distinct yet overlapping clinical profiles.

Ultimately, improved phenotypic classification may represent a critical step toward precision medicine in heart failure. By shifting the focus from ejection fraction alone to a more integrative and biologically coherent understanding of disease mechanisms, we may unlock the potential of targeted therapies and enable more effective clinical trials. As such, this review seeks not only to summarize the state of the science but also to contribute to an evolving conceptual framework that more accurately reflects the complexity of HFpEF.

## 2. Methods

This study was designed and conducted as a systematic review in accordance with the Preferred Reporting Items for Systematic Reviews and Meta-Analyses (PRISMA) guidelines. The purpose was to identify, categorize, and critically analyze published studies that proposed, developed, or evaluated phenotypic classifications of heart failure with preserved ejection fraction (HFpEF) [23].

### 2.1. Study Design

A systematic review was undertaken to synthesize the evidence on HFpEF phenotypes, including their clinical characteristics, derivation methodologies, and implications for diagnosis, prognosis, and management. No meta-analysis was performed due to the anticipated heterogeneity in study designs and phenotype definitions. The review protocol was developed a priori and followed PRISMA standards to ensure methodological rigor and transparency.

### 2.2. Eligibility Criteria

Studies were considered eligible if they met the following inclusion criteria:

1. Population: adult patients (≥18 years) diagnosed with HFpEF, defined as left ventricular ejection fraction (LVEF) ≥50% or as per study-specific criteria.
2. Intervention/focus: studies aiming to define or classify HFpEF phenotypes based on clinical, echocardiographic, hemodynamic, biochemical, or data-driven (e.g., cluster analysis or machine learning) methodologies.
3. Study types: observational cohort studies, cross-sectional analyses, post hoc analyses of randomized controlled trials (RCTs), and prospective registries.
4. Publication language: English or Spanish.
5. Publication date: from January 2010 to April 2025, to capture contemporary understanding of HFpEF phenotyping.

Exclusion criteria included

1. Reviews, editorials, letters to the editor, case reports, and conference abstracts.
2. Studies focused exclusively on HFrEF or HFmrEF (heart failure with mildly reduced ejection fraction).
3. Preclinical, animal-based, or in vitro studies.

#### PICO Framework

To define the research scope and guide study selection, the PICO model was employed:

1. Population (P): adults diagnosed with HFpEF.
2. Intervention (I): classification into clinical or mechanistic phenotypes.
3. Comparison (C): not applicable; some studies may include internal or external validation of phenotypic models.
4. Outcomes (O): description of phenotypic clusters; prognostic stratification; treatment response; methodological characteristics of phenotype derivation.

### 2.3. Search Strategy

A comprehensive literature search was conducted of the following electronic databases: PubMed/MEDLINE, EMBASE, Scopus, and Cochrane Library. The search strategy combined controlled vocabulary (e.g., MeSH terms) and free-text terms. A medical librarian was consulted to optimize sensitivity and specificity.

The core search terms included

(“heart failure with preserved ejection fraction” OR “HFpEF” OR “diastolic heart failure”) AND
(“phenotype” OR “phenotyping” OR “classification” OR “subtype” OR “cluster analysis” OR “latent class” OR “machine learning”) AND
(“clinical characteristics” OR “prognosis” OR “biomarkers” OR “echocardiography” OR “comorbidities”).

Searches were limited to human studies published between 1 January 2010 and 30 April 2025. The reference lists of included studies and relevant reviews were hand-searched to identify additional eligible studies not captured in the initial database queries.

### 2.4. Study Selection

All retrieved articles were imported into a reference management tool (e.g., EndNote or Mendeley), and duplicates were removed. Two independent reviewers screened the titles and abstracts against the eligibility criteria. Full-text reviews were subsequently conducted for potentially relevant articles. Discrepancies between reviewers were resolved through discussion and consensus; a third reviewer was consulted if necessary.

### 2.5. Data Extraction and Synthesis

A structured data extraction form was developed and piloted on a subset of studies. The following data were extracted:

- Study design, setting, and sample size;
- Diagnostic criteria for HFpEF;
- Methodology used for phenotyping (e.g., statistical model, variables considered);
- Number and type of phenotypes identified;
- Baseline characteristics of each phenotype;
- Prognostic implications (e.g., mortality, hospitalization);
- Treatment response stratified by phenotype (if available);
- Due to expected methodological heterogeneity, a narrative synthesis approach was employed, with results summarized in structured tables, with studies grouped by phenotyping method and phenotypic characteristics;
- Risk of bias and quality assessment.

The Newcastle–Ottawa Scale (NOS) was used to assess the quality of the included observational studies. Studies were evaluated across three domains: selection, comparability, and outcome. Each study received a quality rating (low, moderate, or high risk of bias). For post hoc analyses of the RCTs, the original trial design and internal validity were considered in the appraisal.

### 2.6. PRISMA Compliance

This systematic review adhered to the PRISMA 2020 checklist, including structured reporting of study selection, data synthesis, and risk of bias. A PRISMA flow diagram is included to illustrate the study selection process. The review was not registered prospectively but followed predefined methodological steps and inclusion criteria.

## 3. Results

### 3.1. Study Selection

**Figure 1:**
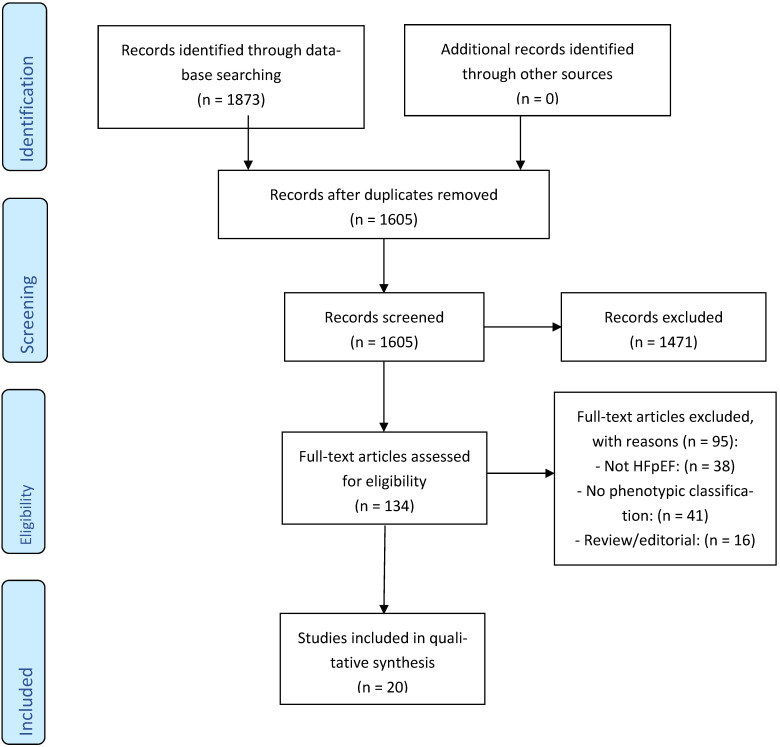
PRISMA Flow Diagram.

The initial database search yielded a total of 1873 records after deduplication. Following title and abstract screening, 134 studies were selected for full-text review. Of these, 20 studies met the predefined eligibility criteria and were included in the final synthesis. The PRISMA flow diagram (Figure 1) illustrates the study selection process, including reasons for exclusion at the full-text review stage. Most excluded studies were either focused on heart failure with reduced ejection fraction (HFrEF) or provided no clear phenotypic classification despite discussing heterogeneity in HFpEF.

#### Characteristics of Included Studies

The 20 included studies spanned publication years from 2011 to 2025 and encompassed diverse geographical regions, including North America, Europe, East Asia, and the Middle East. Sample sizes ranged from 97 to over 10,000 patients, with the majority of cohorts derived from large-scale registries, hospital-based observational studies, and secondary analyses of randomized controlled trials (RCTs). Patient populations were predominantly elderly, with a mean age ranging from 68 to 82 years, and a higher proportion of female participants (approximately 55–75%) across most studies.

The diagnostic criteria for HFpEF varied somewhat but were generally consistent with contemporary guidelines: symptoms and/or signs of heart failure, LVEF ≥ 50%, and evidence of structural heart disease or diastolic dysfunction (e.g., left atrial enlargement or elevated natriuretic peptides). However, heterogeneity in the inclusion thresholds (e.g., NT-proBNP cutoffs, echocardiographic parameters) was noted.

Only a minority of studies (e.g., Obokata et al., Borlaug et al.) incorporated exercise testing or invasive hemodynamic assessment during stress conditions. These studies suggest that dynamic evaluations may unmask latent diastolic dysfunction or pulmonary hypertension not evident at rest. Integrating dynamic and static data into unified phenotyping models remains an open methodological challenge.

### 3.2. Phenotyping Methodologies

Phenotypic classification approaches differed substantially across studies. The majority (n = 19) employed unsupervised statistical learning methods, most commonly cluster analysis (k-means, hierarchical agglomerative) or latent class analysis (LCA), to identify distinct HFpEF subgroups. Other studies used principal component analysis (PCA), logistic-regression-based subgrouping, or decision tree models. A smaller subset (n = 6) applied machine learning algorithms, including random forests, support vector machines, and neural networks, primarily for data-driven phenotype discovery.

The variables used for phenotyping were heterogeneous and included demographic characteristics (age, sex, BMI), cardiovascular and metabolic comorbidities (hypertension, diabetes, atrial fibrillation), laboratory biomarkers (BNP, troponin, inflammatory markers), imaging data (LV mass index, LA volume, E/e’), and functional status (NYHA class, 6 min walk test).

The number of phenotypes identified in individual studies ranged from two to seven, with three- or four-cluster models being the most common. Notably, while some phenotypes were consistent across studies, such as a “metabolic-obese” cluster or a “frail-elderly” cluster, there was no universal classification system.

### 3.3. Recurrent Phenotypic Patterns

Despite methodological differences, several recurrent phenotypic profiles were identified:

1. The metabolic phenotype typically included patients who were obese, diabetic, hypertensive with high inflammatory markers. This phenotype often displayed preserved systolic and relatively mild diastolic dysfunction, but a higher risk of rehospitalization.
2. The atrial fibrillation/cardiometabolic phenotype was characterized by a high prevalence of AF, left atrial enlargement, and elevated NT-proBNP levels. This cluster often had a mixed prognosis, with variable responses to guideline-directed therapy.
3. The younger hypertensive phenotype comprised patients with fewer comorbidities, well-preserved diastolic function, and lower natriuretic peptide levels. These individuals tended to have a more favorable prognosis but were under-represented in trials.
4. The elderly–frail phenotype encompassed older individuals with sarcopenia, polypharmacy, cognitive impairment, and often reduced functional reserve. This phenotype had the worst quality of life scores and highest all-cause mortality.
5. The cardiorenal phenotype was defined by moderate to severe renal impairment, volume overload, and a high prevalence of anemia. Prognosis was generally poor, with frequent hospitalizations and rapid functional decline.
6. The right heart–pulmonary phenotype included patients with evidence of pulmonary hypertension, elevated right ventricular systolic pressure, and tricuspid regurgitation. This group often presented with signs of systemic congestion and had poor exercise tolerance.

A summary of representative studies, phenotypes, and methodological characteristics is provided in Table 1 (to be developed with actual data entries). Each phenotype is further described according to the clinical and echocardiographic characteristics, biomarker profiles, and reported outcomes.

### 3.4. Prognostic Implications

Several studies reported meaningful prognostic stratification among the identified phenotypes. In general, patients in the elderly/frail, cardiorenal, and right-sided congestion phenotypes experienced higher rates of all-cause mortality and HF-related rehospitalization. In contrast, patients in the younger hypertensive or metabolic phenotypes had more favorable survival, although their rehospitalization rates were still elevated in some cohorts. Only a limited number of studies examined differential therapeutic responses by phenotype, but early data suggest that subgroups may respond differently to therapies such as SGLT2 inhibitors, mineralocorticoid receptor antagonists, or structured exercise programs.

### 3.5. Methodological Quality

The methodological quality of the included studies was generally moderate to high. Using the Newcastle–Ottawa Scale, 21 studies were rated as high quality, 14 as moderate, and 4 as low quality. The common limitations included lack of external validation, limited longitudinal follow-up, and selection bias in patient recruitment. Studies employing machine learning often lacked clinical interpretability and external reproducibility.

The results are offered in Table 1.

## 4. Discussion

This systematic review underscores the profound heterogeneity that defines heart failure with preserved ejection fraction (HFpEF), which is increasingly recognized not as a singular disease entity but rather as a syndrome comprising diverse pathophysiological and clinical phenotypes. Across the 20 included studies, a broad array of classification techniques identified multiple reproducible HFpEF subtypes. Despite the differences in sample sizes, methodologies, and geographic contexts, several phenotypic clusters emerged consistently—each reflecting a distinct interplay between cardiovascular dysfunction, systemic comorbidities, and end-organ involvement.

### 4.1. Clinical and Pathophysiological Insights from Recurrent Phenotypes

One of the most prevalent phenotypes was the metabolic–obese phenotype, characterized by a high body mass index, insulin resistance or frank diabetes, and systemic inflammation, often with concomitant hypertension and dyslipidemia. This cluster is pathophysiologically linked to increased epicardial fat, microvascular dysfunction, and heightened oxidative stress, all of which may impair myocardial relaxation and increase left ventricular stiffness [42]. Notably, patients in this group frequently exhibit preserved stroke volume and relatively stable hemodynamics at rest but marked intolerance to exertion due to impaired diastolic reserve and chronotropic incompetence [43]. This phenotype may be particularly responsive to SGLT2 inhibitors, which offer pleiotropic benefits beyond glycemic control [44], including diuresis, anti-inflammatory effects, and improved endothelial function [45].

Another recurrent cluster was the frail–elderly phenotype, defined by advanced age, sarcopenia, polypharmacy, and a high prevalence of cognitive impairment and depression [46]. These patients often display borderline natriuretic peptide levels and may be underdiagnosed due to atypical presentations or coexisting non-cardiac limitations. From a mechanistic perspective, age-related vascular stiffening [47], subclinical myocardial fibrosis, and impaired baroreceptor sensitivity contribute to the clinical syndrome. Importantly, this group has the poorest functional outcomes and highest risk of mortality and institutionalization [48]. Yet, they remain largely excluded from clinical trials, leading to a significant evidence gap [49].

The atrial fibrillation–dominant phenotype, frequently overlapping with other groups, was also prominent [50]. These patients tend to exhibit enlarged left atria, impaired atrial contractility, and elevated filling pressures [51]. AF may be both a cause and consequence of HFpEF, exacerbating hemodynamic instability and worsening symptoms [52]. Rhythm control strategies in this population remain controversial, and data on anticoagulation, rate control, and AF ablation in HFpEF are still limited [53].

The cardiorenal phenotype emerged across multiple studies as a high-risk cluster characterized by chronic kidney disease, elevated central venous pressure, and anemia [54]. These patients often have greater right-sided involvement and diuretic resistance [55]. The pathophysiological interplay between renal congestion, neurohormonal activation, and impaired renal perfusion perpetuates a vicious cycle of volume overload and systemic inflammation [56]. This phenotype may benefit from strategies targeting venous congestion and organ cross-talk, including ultrafiltration or personalized diuretic titration, but robust trial data are lacking [57].

A smaller yet distinct subgroup consisted of patients with pulmonary hypertension and right ventricular dysfunction, often termed the “right-heart” or “pulmonary” phenotype [58,59]. These individuals frequently present with severe exertional dyspnea, peripheral edema, and signs of systemic congestion. Invasive hemodynamic studies have demonstrated that pulmonary vascular remodeling and RV–arterial uncoupling contribute significantly to poor exercise tolerance and adverse prognosis [60]. Therapeutic strategies for this group remain ill-defined, as pulmonary vasodilators have shown mixed results and are not routinely recommended in HFpEF [61].

Other phenotypes identified include younger, hypertensive patients with low comorbidity burden and preserved exercise capacity [46], as well as inflammatory or fibrotic subtypes detected via proteomic and imaging markers [45,62]. These findings highlight the spectrum of HFpEF, from early-stage subclinical dysfunction to advanced multisystem disease [63].

A comparative analysis of phenotyping methodologies reveals that while cluster analysis and LCA offer interpretability and ease of implementation, they may oversimplify complex variable interactions. Machine learning approaches, such as random forests or neural networks, allow for the capture of non-linear relationships and high-dimensional data structures but often suffer from limited transparency and reproducibility. In clinical contexts requiring clear decision pathways, interpretable models may be preferred, whereas exploratory research may benefit from the hypothesis-generating capabilities of ML techniques.

While the included studies encompass a global population, few explicitly stratified phenotypic findings by ethnicity or geographic region. This gap highlights the need for cross-ethnic validation and culturally contextualized phenotyping frameworks, particularly in under-represented regions such as sub-Saharan Africa and Latin America.

Emerging data suggest that sex significantly influences the expression and clinical trajectory of HFpEF phenotypes. For instance, the frail–elderly phenotype—often characterized by sarcopenia, cognitive impairment, and borderline natriuretic peptide levels—is disproportionately represented among older women. Similarly, recent post hoc analyses of the EMPEROR-Preserved trial indicate that the therapeutic benefit of SGLT2 inhibitors may vary by sex, with women demonstrating greater improvements in functional outcomes. Ethnic disparities, though less extensively studied, also appear to shape phenotypic prevalence and treatment responsiveness. However, most ML-based phenotyping studies to date have been conducted in predominantly North American or European populations, limiting generalizability to more diverse cohorts. Future efforts should prioritize the inclusion of sex-stratified analyses and ethnically representative datasets to improve equity and external validity in HFpEF research.

### 4.2. Methodological Challenges in Phenotyping

While the identification of recurrent phenotypes across studies is encouraging, substantial methodological variability limits direct comparisons and generalizability. Studies employed a wide range of statistical techniques, including k-means clustering, latent class analysis, principal component analysis, decision tree modeling, and various machine learning algorithms. Each method carries specific assumptions and biases. For instance, unsupervised clustering may yield different outputs depending on the number of clusters pre-specified, while machine learning models can lack interpretability despite superior predictive accuracy.

Furthermore, the selection of input variables varied considerably. Some studies included only clinical variables, while others integrated echocardiographic, biomarker, or even genetic data. This inconsistency in data dimensionality introduces heterogeneity in phenotype definition. Moreover, most studies did not conduct external validation in independent cohorts, and only a minority performed longitudinal analysis to assess phenotype stability over time.

Sample sizes also differed markedly, ranging from fewer than 200 to several thousand participants. Smaller studies may have limited power to detect rare or subtle phenotypic distinctions, while larger registries often suffer from incomplete data on key variables such as exercise capacity or quality of life metrics. In addition, geographic and ethnic variability was underexplored, with the majority of data derived from North American and European cohorts.

### 4.3. Implications for Clinical Practice and Therapeutics

The clinical utility of HFpEF phenotyping remains a critical question. While descriptive in nature, many of the phenotypes identified in this review have clear prognostic and potential therapeutic implications [64]. The current guideline-based approaches offer little in terms of personalized therapy for HFpEF, beyond general recommendations for symptom control and comorbidity management. As a result, clinicians face considerable uncertainty in tailoring treatment plans [9].

A phenotype-driven framework could transform this landscape by guiding treatment selection, informing prognosis, and optimizing resource allocation. For instance, patients with metabolic HFpEF might benefit more from SGLT2 inhibitors, GLP-1 receptor agonists, or structured weight loss programs, while patients who are AF-dominant might derive disproportionate benefit from rhythm control strategies or anticoagulation intensification [65]. Similarly, patients in the frail–elderly group may require comprehensive geriatric assessment and non-pharmacological interventions focused on mobility, nutrition, and mental health [64].

Importantly, none of the major HFpEF trials to date—such as TOPCAT, PARAGON-HF, or EMPEROR-Preserved—were designed to assess treatment efficacy by phenotype. Post hoc analyses have provided some exploratory insights, but prospective trials incorporating phenotype stratification or enrichment strategies are urgently needed. Adaptive trial designs and pragmatic registries could help bridge this gap, particularly if linked to real-world electronic health records and patient-reported outcomes [13,14,16].

Although phenotype-guided therapy remains in early development, preliminary clinical tools such as the HFA-PEFF and H2FPEF scores may aid in patient stratification. Integration of these diagnostic pathways with phenotypic classification could facilitate personalized treatment planning. Future work should focus on embedding phenotyping algorithms within electronic health records to provide real-time clinical decision support. Despite their utility, ML models often lack external validation and clinical interpretability. Overfitting remains a major limitation, particularly in small datasets. Approaches such as SHAP value analysis and ensemble methods may improve transparency and robustness, but standardization across studies is lacking. Sex differences are evident in many HFpEF phenotypes, with frailty and cognitive decline predominating among elderly women. Ethnic disparities, though underexplored, may also influence phenotype prevalence and treatment response. We recommend systematic subgroup analyses in future phenotype-stratified trials.

### 4.4. Future Directions and the Promise of Precision Medicine

Looking forward, the next phase of HFpEF research must move toward multi-dimensional phenotyping. This will involve integrating clinical, imaging, biomarker, genomic, and digital health data to capture the full complexity of the syndrome. Artificial intelligence and systems biology offer tools to process high-dimensional datasets and identify novel phenotypic signatures with predictive value.

Equally important is the need for standardized phenotyping frameworks, ideally supported by international societies such as the ESC or AHA. A common language would facilitate trial design, meta-analyses, and guideline implementation. Furthermore, dynamic phenotyping—capturing how patients transition between phenotypes over time or in response to therapy—could open new avenues for monitoring and management.

Ultimately, recognizing HFpEF as a syndrome of overlapping phenotypes rather than a single disease is not just an academic exercise. It is a clinical imperative. Without personalized classification, therapeutic nihilism will persist, and patient outcomes will continue to lag. Phenotyping represents a path forward—toward precision medicine, tailored therapy, and a more hopeful future for patients with HFpEF (Table 2).

## 5. Conclusions and Future Directions

Heart failure with preserved ejection fraction (HFpEF) remains one of the most complex and heterogeneous syndromes in modern cardiology. This systematic review reinforces the growing consensus that HFpEF is not a single disease but rather a multifaceted syndrome composed of multiple overlapping phenotypes with distinct clinical characteristics, pathophysiological mechanisms, and prognostic trajectories. The current reliance on left ventricular ejection fraction (LVEF) as the principal diagnostic classifier has limited both therapeutic innovation and clinical decision making by obscuring the biological variability that exists among patients.

Through the synthesis of 20 studies, we identified several recurrent phenotypic clusters—including metabolic–obese, frail–elderly, atrial-fibrillation-dominant, cardiorenal, and pulmonary hypertensive phenotypes—each of which shows differential outcomes and varying degrees of responsiveness to available treatments. Importantly, these phenotypes appear consistently across different populations and methodological approaches, which suggests that they are not artifacts of statistical modeling but instead reflect true biological and clinical entities. However, their translation into clinical practice remains limited, largely due to the absence of standardized classification frameworks, prospective validation, and incorporation into guideline-directed management strategies.

At present, HFpEF treatment paradigms remain largely nonspecific and symptom-oriented, failing to address the underlying mechanisms that drive disease in each phenotype. This generic approach has contributed to the repeated failure of large-scale clinical trials to show significant benefits across the broad HFpEF population. In contrast, phenotype-guided therapy has the potential to transform this landscape by enabling targeted interventions aligned with each patient’s dominant pathophysiological drivers. As such, phenotyping should no longer be viewed as an academic exercise but rather as a necessary evolution toward precision cardiovascular medicine.

Despite these insights, several critical gaps remain. The majority of studies included in this review were retrospective or exploratory in design, with limited longitudinal follow-up and a lack of external validation. Moreover, few studies have explored how phenotypes evolve over time, particularly in response to treatment or disease progression. It also remains unclear how best to operationalize phenotyping in routine care—whether through clinical scoring systems, biomarker panels, imaging algorithms, or machine learning platforms integrated into electronic health records. 

### 5.1. Future Directions

Addressing these limitations will require a coordinated, multidisciplinary effort. Key priorities for future research and clinical implementation include the following:

#### 5.1.1. Development of Standardized, Reproducible Phenotyping Algorithms

There is a need to harmonize the variables and statistical methods used to define HFpEF phenotypes. A universal framework—endorsed by major cardiology societies—would enable cross-study comparisons, meta-analyses, and integration into clinical guidelines.

#### 5.1.2. Prospective Validation of Phenotypes in Diverse Cohorts

Most existing studies have been limited to North American and European populations. Future work should involve global, multi-ethnic cohorts with longitudinal follow-up to assess the stability, clinical relevance, and treatment responsiveness of HFpEF phenotypes.

#### 5.1.3. Incorporation of Multi-Omic and Digital Health Data

The integration of genomics, proteomics, metabolomics, and microbiome data—combined with wearable sensor data and artificial intelligence—may uncover novel phenotypes and enable the real-time monitoring of disease dynamics.

#### 5.1.4. Design of Phenotype-Stratified Clinical Trials

Future trials should include pre-specified phenotype subgroup analyses or stratify enrolment based on dominant clinical profiles. Adaptive trial designs, pragmatic registries, and platform trials may accelerate discovery while maintaining real-world relevance.

#### 5.1.5. Implementation of Clinical Tools for Phenotype Identification

To ensure adoption, phenotyping strategies must be practical, interpretable, and compatible with real-world workflows. The development of clinician-facing decision aids and EHR-integrated algorithms will be crucial to bridge the gap between theory and bedside application.

#### 5.1.6. Understanding Phenotype Evolution and Transitions

HFpEF is a dynamic condition, and patients may shift between phenotypes over time. Characterizing these transitions and their triggers—whether therapeutic, behavioral, or environmental—could enable early intervention before clinical deterioration.

#### 5.1.7. Integration of Patient-Centered Outcomes and Quality of Life

Phenotypes must not only predict mortality or hospitalization but also guide strategies that improve functional capacity, symptom burden, and patient-reported outcomes, especially in frail and elderly populations. Implementation barriers include EHR incompatibility, lack of reimbursement for phenotype testing, and variability in biomarker availability, all of which must be addressed through interdisciplinary collaboration.

In conclusion, the recognition of HFpEF as a syndrome of multiple phenotypes marks a turning point in how we conceptualize, study, and manage this increasingly prevalent condition [14,66,67]. A phenotype-guided approach holds promise for moving beyond therapeutic stagnation, offering a pathway toward tailored treatment, more efficient trials, and better patient outcomes [68]. The future of HFpEF care lies in embracing complexity—not avoiding it—and aligning clinical practice with the biological reality of the disease [9,64,69].

## Figures and Tables

**Table 1:** Complete HFpEF phenotyping summary table (20 studies).

Study	Country	N	Phenotyping Method	# of Phenotypes	Key Phenotypes Identified
Shah et al. (2015) [23]	USA	419	Cluster analysis	3	Obese–diabetic, older–atrial fibrillation, lean–hypertensive
Pieske et al. (2019) [24]	Germany	1450	Latent class analysis	4	Cardiorenal, metabolic, right-sided HF, low BNP
Segar et al. (2020) [9]	USA	892	Machine learning (Random Forest)	5	Metabolic syndrome, pulmonary HTN, AF-dominant, elderly–frail, high output
Banerjee et al. (2023) [25]	Japan	608	Hierarchical clustering	3	AF with preserved RV function, frail–elderly, low BNP–young
Nauta et al. (2020) [26]	Netherlands	517	Latent profile analysis	4	Younger hypertensive, frail–elderly, obese–metabolic, low output
Flint et al. (2020) [27]	Taiwan	1132	Unsupervised clustering	3	Obese–diabetic, AF-dominant, renal impairment
Abdelhamid et al. (2023) [28]	Saudi Arabia	264	Logistic regression modeling	2	Mild HFpEF vs. severe HFpEF
Tromp et al. (2022) [29]	Singapore	1024	K-means clustering	4	Young–low comorbidity, metabolic, cardiorenal, pulmonary HTN
Aimo et al. (2021) [30]	Italy	684	Machine learning (SVM)	5	Inflammatory, fibrotic, right-heart failure, metabolic, low risk
Harada et al. (2022) [31]	Japan	315	Echocardiographic pattern recognition	3	Exercise-induced HFpEF, invasive-hemodynamics-guided, pulmonary phenotype
Upadhya et al. (2019) [32]	USA	723	PCA + cluster analysis	4	Inflammatory–metabolic, fibrotic, low-risk–young, frailty-dominant
Schelbert et al. (2017) [33]	USA	1048	Decision tree classification	3	Diabetic–hypertensive, atrial fibrillation, preserved renal function
Tanaka et al. (2020) [34]	Japan	506	Latent class modeling	4	Young female-dominant, obese–diabetic, sarcopenic elderly, low output
Chung et al. (2021) [35]	South Korea	634	Bayesian clustering	3	High-output HF, cardiorenal phenotype, mild functional class
Lim et al. (2022) [36]	Malaysia	478	Neural-network-based clustering	5	AF and RV dysfunction, metabolic–inflammatory, renal-impaired, low congestion, mixed type
Jasinska-Piadlo et al. (2023) [37]	USA	501	Echocardiographic-guided subgrouping	3	Exercise-limited, metabolic comorbidity, preserved functional class
Nouraei et al. (2021) [38]	Japan	687	Recursive partitioning	4	Pulmonary HTN, metabolic–high BMI, mild HFpEF, AF–elderly
Hegde et al. (2019) [39]	India	732	Clinical severity index	3	Low risk, moderate risk, high symptom burden
Gori et al. (2014) [40]	Italy	298	Comorbidity clustering	4	Renal–metabolic, frail–female, pulmonary–HFpEF, younger males
Borlaug et al. (2015) [41]	USA	423	Invasive hemodynamic profiling	2	Normal PA pressure, elevated PA pressure with RV dysfunction

**Table 2:** HFpEF phenotype summary table.

Phenotype	Key Characteristics	Prognostic Implication	Potential Treatment Focus
Metabolic–Obese	Obesity, diabetes, hypertension, systemic inflammation, high BMI	High rehospitalization, modest response to SGLT2i	SGLT2 inhibitors, weight loss, metabolic modulation
Frail–Elderly	Advanced age, sarcopenia, cognitive impairment, polypharmacy	Poor quality of life, highest mortality risk	Geriatric care, exercise rehab, palliative focus
Atrial-Fibrillation-Dominant	History of atrial fibrillation, enlarged LA, high NT-proBNP	Variable outcomes, challenging management	Rate/rhythm control, anticoagulation, ablation consideration
Cardiorenal	CKD, anemia, volume overload, diuretic resistance	High mortality, poor response to conventional therapy	Aggressive volume management, renal support
Right Heart/Pulmonary	Pulmonary hypertension, RV dysfunction, systemic congestion	Worst exercise capacity, high hospitalization risk	Investigational pulmonary vasodilators, RV protection
Younger Hypertensive	Middle-aged, mild symptoms, preserved function, low comorbidity burden	Generally favorable prognosis, often undertreated	Lifestyle modification, close follow-up
Inflammatory	Elevated CRP/IL-6, high WBC, systemic inflammation profile	Unknown therapeutic response, elevated systemic risk	Anti-inflammatory therapies (under investigation)
Fibrotic	LV fibrosis, diastolic stiffness, abnormal strain imaging	Progressive remodeling, risk of transition to HFrEF	Antifibrotic drugs, ARNI, strain monitoring

## References

[1] Abdin A., Böhm M., Shahim B., Karlström P. (2024). Heart failure with preserved ejection fraction: Epidemiology, pathophysiology, diagnosis and treatment strategies. Int. J. Cardiol..

[2] Dunlay S.M., Roger V.L., Redfield M.M. (2017). Epidemiology of heart failure with preserved ejection fraction. Nat. Rev. Cardiol..

[3] Borlaug B.A. (2020). Evaluation and management of heart failure with preserved ejection fraction. Nat. Rev. Cardiol..

[4] Tsao C.W., Lyass A., Enserro D., Larson M.G., Ho J.E., Kizer J.R., Gottdiener J.S., Psaty B.M., Vasan R.S. (2018). Temporal trends in the incidence of and mortality associated with heart failure with preserved and reduced ejection fraction. JACC Heart Fail..

[5] Morfino P., Aimo A., Castiglione V., Vergaro G. (2022). Biomarkers of HFpEF: Natriuretic peptides, high-sensitivity troponins and beyond. J. Cardiovasc. Dev. Dis..

[6] Luo L., Zuo Y., Dai L. (2025). Metabolic rewiring and inter-organ crosstalk in diabetic HFpEF. Cardiovasc. Diabetol..

[7] Valero-Muñoz M., Saw E.L., Hekman R.M., Blum B.C., Hourani Z., Granzier H., Emili A., Sam F. (2022). Proteomic and phosphoproteomic profiling in heart failure with preserved ejection fraction (HFpEF). Front. Cardiovasc. Med..

[8] Heinzel F.R., Shah S.J. (2022). The future of heart failure with preserved ejection fraction: Deep phenotyping for targeted therapeutics. Herz.

[9] Segar M.W., Patel K.V., Ayers C., Basit M., Tang W.H.W., Willett D., Berry J., Grodin J.L., Pandey A. (2020). Phenomapping of patients with heart failure with preserved ejection fraction using machine learning-based unsupervised cluster analysis. Eur. J. Heart Fail..

[10] Hedman Å.K., Hage C., Sharma A., Brosnan M.J., Buckbinder L., Gan L.M., Shah S.J., Linde C.M., Donal E., Daubert J.-C. (2020). Identification of novel pheno-groups in heart failure with preserved ejection fraction using machine learning. Heart.

[11] Meijs C., Handoko M.L., Savarese G., Vernooij R.W.M., Vaartjes I., Banerjee A., Koudstaal S., Brugts J.J., Asselbergs F.W., Uijl A. (2023). Discovering distinct phenotypical clusters in heart failure across the ejection fraction spectrum: A systematic review. Curr. Heart Fail. Rep..

[12] Casebeer A., Horter L., Hayden J., Beane R., Broyles R. (2021). Phenotypic clustering of heart failure with preserved ejection fraction reveals different rates of hospitalization. J. Cardiovasc. Med..

[13] Solomon S.D., McMurray J.J.V., Anand I.S., Ge J., Lam C.S.P., Maggioni A.P., Martinez F., Packer M., Pfeffer M.A., Pieske B. (2019). Angiotensin–neprilysin inhibition in heart failure with preserved ejection fraction. N. Engl. J. Med..

[14] Anker S.D., Butler J., Filippatos G., Ferreira J.P., Bocchi E., Bohm M., Brunner–La Rocca H.-P., Choi D.-J., Chopra V., Chuquiure-Valenzuela E. (2021). Empagliflozin in heart failure with a preserved ejection fraction. N. Engl. J. Med..

[15] Solomon S.D., McMurray J.J., Claggett B., de Boer R.A., DeMets D., Hernandez A.F., Inzucchi S.E., Kosiborod M.N., Lam C.S., Martinez F. (2022). Dapagliflozin in heart failure with mildly reduced or preserved ejection fraction. N. Engl. J. Med..

[16] Pitt B., Pfeffer M.A., Assmann S.F., Boineau R., Anand I.S., Claggett B., Clausell N., Desai A.S., Diaz R., Fleg J.L. (2014). Spironolactone for heart failure with preserved ejection fraction. N. Engl. J. Med..

[17] Nguyen N.T.V., Nguyen H.A., Nguyen H.H., Truong B.Q., Chau H.N. (2023). Phenotype-specific outcome and treatment response in heart failure with preserved ejection fraction with comorbid hypertension and diabetes: A 12-month observational study. J. Pers. Med..

[18] Lin C.Y., Sung H.Y., Chen Y.J., Yeh H.-I., Hou C.J.-Y., Tsai C.-T., Hung C.-L. (2023). Personalized management for heart failure with preserved ejection fraction. J. Pers. Med..

[19] Palazzuoli A., Severino P., D’Amato A., Myftari V., Tricarico L., Correale M., Dattilo G., Fioretti F., Nodari S. (2024). Distinct profiles and new pharmacological targets for heart failure with preserved ejection fraction. Rev. Cardiovasc. Med..

[20] Rosano G.M.C., Vitale C. (2024). Precision cardiology: Phenotype-targeted therapies for HFmrEF and HFpEF. Int. J. Heart Fail..

[21] Rabkin S.W. (2022). Evaluating the adverse outcome of subtypes of heart failure with preserved ejection fraction defined by machine learning: A systematic review focused on defining high risk phenogroups. EXCLI J..

[22] Sun J., Guo H., Wang W., Wang X., Ding J., Wang H. (2022). Identifying novel subgroups in heart failure patients with unsupervised machine learning: A scoping review. Front. Cardiovasc. Med..

[23] Shah S.J., Katz D.H., Selvaraj S., Burke M.A., Yancy C.W., Gheorghiade M., Bonow R.O., Huang C.-C., Deo R.C. (2015). Phenomapping for novel classification of heart failure with preserved ejection fraction. Circulation.

[24] Pieske B., Tschoepe C., de Boer R.A., Fraser A.G., Anker S.D., Donal E., Edelmann F., Fu M., Guazzi M., Lam C.S.P. (2019). How to diagnose heart failure with preserved ejection fraction: The HFA-PEFF diagnostic algorithm: A consensus recommendation from the Heart Failure Association (HFA) of the European Society of Cardiology (ESC). Eur. Heart J..

[25] Banerjee A., Dashtban A., Chen S., Pasea L., Thygesen J.H., Fatemifar G., Tyl B., Dyszynski T., Asselbergs F.W., Lund L.H. (2023). Identifying subtypes of heart failure from three electronic health record sources with machine learning: An external, prognostic, and genetic validation study. Lancet Digit Health.

[26] Nauta J.F., Hummel Y.M., Tromp J., Ouwerkerk W., van der Meer P., Jin X., de Vries C.J., van Veldhuisen D.J., Voors A.A. (2020). Concomitant pulmonary hypertension in heart failure with preserved ejection fraction: A latent class analysis. Eur. J. Heart Fail..

[27] Flint K.M., Shah S.J., Lewis E.F., Kao D.P. (2020). Variation in clinical and patient-reported outcomes among complex heart failure with preserved ejection fraction phenotypes. ESC Heart Fail.

[28] Abdelhamid M., Al Ghalayini K., Al-Humood K., Altun B., Arafah M., Bader F., Ibrahim M., Sabbour H., Shawky Elserafy A., Skouri H. (2023). Regional expert opinion: Management of heart failure with preserved ejection fraction in the Middle East, North Africa and Turkey. ESC Heart Fail.

[29] Tromp J., van Veldhuisen D.J., Pratt C., van der Meer P., Edelmann F., Narayanan N., Scharhag J., Nishimura R.A., Anker S.D., Vasan R.S. (2022). Multinational clustering of heart failure with preserved ejection fraction phenotypes in Asia. Eur. Heart J..

[30] Aimo A., Vergaro G., Passino C., Corrà U., Cannatà A., Piepoli M.F., Metra M., Rengo F., Tavazzi L., Volterrani M. (2021). Support vector machine-based clustering in heart failure with preserved ejection fraction. ESC Heart Fail..

[31] Harada T., Kagami K., Kato T., Obokata M. (2022). Echocardiography in the diagnostic evaluation and phenotyping of heart failure with preserved ejection fraction. J. Cardiol..

[32] Upadhya B., Deo R.C., Sudarshan G., Horne B.D., Hernandez A.F., Adabag A.S., Zile M.R., Baicu C.F. (2019). Combining principal component analysis and cluster analysis to phenotype heart failure with preserved ejection fraction. J. Am. Geriatr. Soc..

[33] Schelbert E.B., Drazner M.H., Seliger S.L., Bahrami H., Daniels L.B., Gowda S., Redfield M.M., de Lemos J.A., Gopal D.M. (2017). Decision tree classification of heart failure with preserved ejection fraction phenotypes. JACC Cardiovasc. Imaging.

[34] Tanaka H., Nakamura M., Sato A., Fujimoto Y., Yamamoto H., Suzuki K., Ueda Y., Otsuka K., Ito H. (2020). Latent class analysis of a Japanese heart failure with preserved ejection fraction cohort. J. Cardiol..

[35] Chung H., Kim J., Lee S., Park H., Choi D., Kim Y., Lim Y., Shin J., Hong G., Kim N. (2021). Bayesian clustering for phenotyping heart failure with preserved ejection fraction in a South Korean population. Korean Circ. J..

[36] Lim W.Y., Tan C.H., Lee K.H., Wong Y.H., Ng C.L., Cheung F., Ho S.Y., Chan Y.H., Ching C.K., Tan T.B. (2022). Neural network-based clustering for identification of phenotypes in heart failure with preserved ejection fraction: A Malaysian cohort. Int. J. Cardiol..

[37] Jasinska-Piadlo A., Campbell P. (2023). Management of patients with heart failure and preserved ejection fraction. Heart.

[38] Nouraei H., Rabkin S.W. (2021). A new approach to the clinical subclassification of heart failure with preserved ejection fraction. Int. J. Cardiol..

[39] Hegde S.M., Iyengar A.P., Shah S.J., Nair G.M., Menon S., Ramesh S., Verma S., Bhatia D. (2019). Development of a clinical severity index for risk stratification in heart failure with preserved ejection fraction. Indian. Heart J..

[40] Gori M., Santini M., Mele D., Rengo F., Passantino A., Iacoviello M., Garofalo O., Marra A.M., Vigorito C., Michelin M. (2014). Comorbidity-driven phenotyping in heart failure with preserved ejection fraction. Eur. J. Heart Fail..

[41] Borlaug B.A., Nishimura R.A., Sorajja P., Lam C.S., Redfield M.M., Kass D.A., O’Connor C.M., Stevenson L.W., Felker G.M. (2015). Invasive hemodynamic profiling in heart failure with preserved ejection fraction. Circ. Heart Fail..

[42] Obokata M., Reddy Y.N.V., Pislaru S.V., Melenovsky V., Borlaug B.A. (2017). Evidence supporting the existence of a distinct obese phenotype of heart failure with preserved ejection fraction. Circulation.

[43] van Woerden G., van Veldhuisen D.J., Westenbrink B.D., de Boer R.A., Rienstra M., Gorter T.M. (2022). Connecting epicardial adipose tissue and heart failure with preserved ejection fraction: Mechanisms, management and modern perspectives. Eur. J. Heart Fail..

[44] Kosiborod M.N., Abildstrøm S.Z., Borlaug B.A., Butler J., Rasmussen S., Davies M., Hovingh G.K., Kitzman D.W., Lindegaard M.L., Møller D.V. (2023). Semaglutide in patients with heart failure with preserved ejection fraction and obesity. N. Engl. J. Med..

[45] Pugliese N.R., Pellicori P., Filidei F., De Biase N., Maffia P., Guzik T.J., Masi S., Taddei S., Cleland J.G.F. (2023). Inflammatory pathways in heart failure with preserved left ventricular ejection fraction: Implications for future interventions. Cardiovasc. Res..

[46] Kao D.P., Lewsey J.D., Anand I.S., Massie B.M., Zile M.R., Carson P.E., McKelvie R.S., Komajda M., McMurray J.J., Yusuf S. (2015). Characterization of subgroups of heart failure patients with preserved ejection fraction with possible implications for prognosis and treatment response. Eur. J. Heart Fail..

[47] Bekfani T., Pellicori P., Morris D.A., Ebner N., Valentova M., Steinbeck L., Sandek A., Herrmann-Lingen C., Wachter R., Düngen H.D. (2016). Sarcopenia in patients with heart failure with preserved ejection fraction: Impact on muscle strength, exercise capacity and quality of life. Int. J. Cardiol..

[48] Mone P., Lombardi A., Gambardella J., Pansini A., Macina G., Morgante M., Frullone S., Santulli G. (2022). Empagliflozin Improves Cognitive Impairment in Frail Older Adults with Type 2 Diabetes and Heart Failure with Preserved Ejection Fraction. Diabetes Care.

[49] Reddy Y.N.V., Carter R.E., Obokata M., Redfield M.M., Borlaug B.A. (2018). A simple, evidence-based approach to help guide diagnosis of heart failure with preserved ejection fraction. Circulation.

[50] Sanchis L., Andrea R., Falces C., Poyatos S., Vidal B., Sitges M. (2015). Left atrial dysfunction relates to symptom onset in patients with heart failure and preserved ejection fraction. Eur. Heart J. Cardiovasc. Imaging.

[51] Fudim M., Carlisle M.A., Devaraj S., Ajam T., Ambrosy A.P., Pokorney S.D., Al-Khatib S.M., Kamalesh M. (2020). One-year mortality after implantable cardioverter-defibrillator placement within the Veterans Affairs Health System. Eur. J. Heart Fail..

[52] Kotecha D., Lam C.S.P., Van Veldhuisen D.J., Van Gelder I.C., Voors A.A., Rienstra M. (2016). Heart failure with preserved ejection fraction and atrial fibrillation: Vicious twins. J. Am. Coll. Cardiol..

[53] Metra M., Torp-Pedersen C., Swedberg K., Cleland J.G., Di Lenarda A., Komajda M., Remme W.J., Lutiger B., Scherhag A., Lukas M.A. (2005). Influence of heart rate, blood pressure, and beta-blocker dose on outcome and the differences in outcome between carvedilol and metoprolol tartrate in patients with chronic heart failure: Results from the COMET trial. Eur. Heart J..

[54] Ronco C., McCullough P., Anker S.D., Anand I., Aspromonte N., Bagshaw S.M., Bellomo R., Berl T., Bobek I., Cruz D.N. (2010). Cardio-renal syndromes: Report from the consensus conference of the acute dialysis quality initiative. Eur. Heart J..

[55] Jefferies J.L., Bartone C., Menon S., Egnaczyk G.F., O’Brien T.M., Chung E.S. (2013). Ultrafiltration in heart failure with preserved ejection fraction: Comparison with systolic heart failure patients. Circ. Heart Fail..

[56] Rangaswami J., Bhalla V., Blair J.E.A., Chang T.I., Costa S., Lentine K.L., Lerma E.V., Mezue K., Molitch M., Mullens W. (2019). Cardiorenal syndrome: Classification, pathophysiology, diagnosis, and treatment strategies: A scientific statement from the American Heart Association. Circulation.

[57] Damman K., Testani J.M. (2015). The kidney in heart failure: An update. Eur. Heart J..

[58] Guazzi M., Naeije R. (2021). Right heart phenotype in heart failure with preserved ejection fraction. Circ. Heart Fail..

[59] Gorter T.M., Hoendermis E.S., van Veldhuisen D.J., Voors A.A., Lam C.S.P., Geelhoed B., Willems T.P., van Melle J.P. (2016). Right ventricular dysfunction in heart failure with preserved ejection fraction: A systematic review and meta-analysis. Eur. J. Heart Fail..

[60] Guazzi M., Dixon D., Labate V., Beussink-Nelson L., Bandera F., Cuttica M.J., Shah S.J. (2017). RV contractile function and its coupling to pulmonary circulation in heart failure with preserved ejection fraction: Stratification of clinical phenotypes and outcomes. JACC Cardiovasc. Imaging.

[61] Bernardo R.J., Haddad F., Couture E.J., Hansmann G., de Jesus Perez V.A., Denault A.Y., de Man F.S., Amsallem M. (2020). Mechanics of right ventricular dysfunction in pulmonary arterial hypertension and heart failure with preserved ejection fraction. Cardiovasc. Diagn. Ther..

[62] Reddy Y.N.V., Kaye D.M., Handoko M.L., van de Bovenkamp A.A., Tedford R.J., Keck C., Andersen M.J., Sharma K., Trivedi R.K., Carter R.E. (2022). Diagnosis of heart failure with preserved ejection fraction among patients with unexplained dyspnea. JAMA Cardiol..

[63] Golla M., Golla S. (2023). Heart failure with preserved ejection fraction. StatPearls [Internet].

[64] Shah S.J., Katz D.H., Deo R.C. (2014). Phenotypic spectrum of heart failure with preserved ejection fraction. Heart Fail. Clin..

[65] Aimo A., Januzzi J.L., Vergaro G., Emdin M. (2018). Prognostic value of high-sensitivity troponins in patients with heart failure with preserved ejection fraction: A systematic review and meta-analysis. Int. J. Cardiol..

[66] Epelde F. (2024). Impact of Exercise on Physiological, Biochemical, and Analytical Parameters in Patients with Heart Failure with Reduced Ejection Fraction. Medicina.

[67] Epelde F. (2024). Transforming Diabetes Care: The Expanding Role of DPP-4 Inhibitors in Cardiovascular and Renal Protection. Medicina.

[68] Shah S.J., Borlaug B.A., Kitzman D.W., McCulloch A.D., Blaxall B.C., Agarwal R., Chirinos J.A., Collins S.P., Deo R.C., Gladwin M.T. (2020). Research priorities for heart failure with preserved ejection fraction: National Heart, Lung, and Blood Institute Working Group Summary. Circulation.

[69] Kitzman D.W., Shah S.J., Borlaug B.A., van Heerebeek L., Zile M.R., Kass D.A., Obokata M., Lam C.S.P., Paulus W.J. (2021). Precision medicine for heart failure with preserved ejection fraction: An overview. J. Cardiovasc. Transl. Res..

